# Cellular temperature compensation of sensory receptor neuron responses

**DOI:** 10.1186/1471-2202-14-S1-O11

**Published:** 2013-07-08

**Authors:** Frederic A Roemschied, Monika JB Eberhard, Jan-Hendrik Schleimer, Bernhard Ronacher, Susanne Schreiber

**Affiliations:** 1 Institute for Theoretical Biology, Humboldt-Universität zu Berlin, 10115 Berlin, Germany; 2 Bernstein Center for Computational Neuroscience Berlin, Humboldt-Universität zu Berlin, 10115 Berlin, Germany; 3 Department of Biology, Humboldt-Universität zu Berlin, 10115 Berlin, Germany

## 

Temperature is known to modulate ion channel kinetics and hence also action-potential generation. This poses a challenge for neural systems that need to retain their functionality also under conditions of varying temperature. Multiple strategies to counterbalance the effects of environmental temperature changes exist: mammals keep their body temperature approximately constant, while poikilothermic species need to implement temperature-compensation at the behavioral, systems, or cellular level. While mechanisms of behavioral and systems level have been identified [1], cellular mechanisms of temperature-compensation as well as their associated metabolic cost remain largely unknown.

We investigated the effect of temperature on auditory processing in the grasshopper. We recorded intracellular responses of auditory receptor neurons to auditory broad-band noise stimuli at different intensities at two distinct behaviorally relevant temperatures. Interestingly, we found that changes in temperature did not have large effects on sound-intensity coding in receptor neurons. These neurons constitute the input layer of a feedforward network and hence do not receive network input. We concluded that the observed temperature robustness of receptor-neuron responses must arise from *intrinsic*, network unrelated effects.

In general, the receptor-neuron response is shaped by two processing steps: mechanosensory transduction and spike generation. Both can contribute to temperature compensation. Either both transduction and spike generation are compensated (hypothesis I), or alternatively, their temperature dependencies can cancel each other (hypothesis II).

To test hypothesis I we assumed a temperature-invariant transduction and asked, first, whether temperature-compensation could be achieved for a spike-generating mechanism with realistic temperature dependencies of the ionic conductances. The latter refers, in particular, to increases of gating kinetics by a factor of 2-4 with temperature increments of 10°C (defining a Q10 value of 2-4) as well as modest increases of peak conductances. Second, we explored whether temperature compensation, if achieved cell-intrinsically, compromises the neuronal energy budget. In other words, is temperature robustness metabolically expensive? To address these questions, we varied the temperature dependence of ionic conductances in a conductance-based neuron model. Based on the spike frequency vs. input current (f-I) relation, we estimated the ability of the model neurons to keep a robust firing rate despite changing temperature. Moreover, we computed the average energetic cost per action potential [2]. Using a database modeling approach [3], we performed a systematic sensitivity analysis for firing-rate changes and energetic cost as a function of the temperature dependence of conductance parameters (i.e. Q10 values of transition rates and peak conductances). Our analysis shows that the key parameters determining the robustness of spike generation relate to the temperature-dependence of the model's *potassium *conductances. In contrast, energy consumption is governed by the temperature dependence of the *sodium *conductance. Consequently, a neuron can achieve temperature-compensation of its firing rate without compromising the energy budget.

To constrain hypothesis II, we used the experimentally observed f-I curves in an objective function and inferred the corresponding transduction process for each spike generation in our sensitivity analysis. Our results predict that thermosensitive Transient Receptor Potential (TRP) channels have a role in mechanosensory transduction at the grasshopper tympanum, and therefore motivate further experiments.
